# Resting and TMS-EEG markers of treatment response in major depressive disorder: A systematic review

**DOI:** 10.3389/fnhum.2022.940759

**Published:** 2022-08-04

**Authors:** Rebecca Strafella, Robert Chen, Tarek K. Rajji, Daniel M. Blumberger, Daphne Voineskos

**Affiliations:** ^1^Institute of Medical Science, University of Toronto, Toronto, ON, Canada; ^2^Temerty Centre for Therapeutic Brain Intervention, Campbell Family Mental Health Research Institute, Centre for Addiction and Mental Health, Toronto, ON, Canada; ^3^Krembil Research Institute, Toronto Western Hospital, University Health Network, Toronto, ON, Canada; ^4^Division of Neurology, Department of Medicine, University of Toronto, Toronto, ON, Canada; ^5^Department of Psychiatry, Temerty Faculty of Medicine, University of Toronto, Toronto, ON, Canada; ^6^Toronto Dementia Research Alliance, University of Toronto, Toronto, ON, Canada

**Keywords:** major depressive disorder (MDD), electroencephalography (EEG), transcranial magnetic stimulation (TMS), TMS-EEG, biomarkers, repetitive transcranial magnetic stimulation (rTMS), antidepressant, treatment

## Abstract

Electroencephalography (EEG) is a non-invasive method to identify markers of treatment response in major depressive disorder (MDD). In this review, existing literature was assessed to determine how EEG markers change with different modalities of MDD treatments, and to synthesize the breadth of EEG markers used in conjunction with MDD treatments. PubMed and EMBASE were searched from 2000 to 2021 for studies reporting resting EEG (rEEG) and transcranial magnetic stimulation combined with EEG (TMS-EEG) measures in patients undergoing MDD treatments. The search yielded 966 articles, 204 underwent full-text screening, and 51 studies were included for a narrative synthesis of findings along with confidence in the evidence. In rEEG studies, non-linear quantitative algorithms such as theta cordance and theta current density show higher predictive value than traditional linear metrics. Although less abundant, TMS-EEG measures show promise for predictive markers of brain stimulation treatment response. Future focus on TMS-EEG measures may prove fruitful, given its ability to target cortical regions of interest related to MDD.

## Introduction

Major depressive disorder (MDD) is a leading cause of disability worldwide and is increasing in prevalence (Friedrich, 2017). Unfortunately, little progress has been made in identifying biological indicators of treatment response, and much intervention is *via* trial-and-error. While some possible neurobiological indicators of response have been identified using genetic and imaging studies (reviewed Belmaker, 2008; Kupfer et al., 2012), a less-costly, non-invasive option is electroencephalography (EEG), which indexes neural activity with high temporal resolution (Berger, 1929). EEG has several investigational uses, characterizing cortical activity after perturbation, or reflecting frequency bands associated with specific cognitive patterns (Freeman and Quiroga, 2013). The combination of transcranial magnetic stimulation with EEG (TMS-EEG) has sparked interest as a way to record direct and downstream cortical responses to a targeted magnetic stimulus (Farzan et al., 2016).

EEG provides multiple avenues to identify putative markers differentiating treatment responders and non-responders in MDD. Common interventions for MDD include pharmacotherapy, psychotherapy, and brain stimulation (Voineskos et al., 2020). However, there is a lack of synthesis of evidence in the MDD literature regarding the utility of EEG indices as markers of treatment response. To date, one meta-analysis has focused solely on quantitative EEG to examine markers of treatment response (Widge et al., 2019), but did not find reliable indices or include other types of EEG investigations, such as TMS-EEG. Due to the breadth of EEG markers in the existing literature, there is a need to combine evidence to understand which markers consistently demonstrate the potential for clinical utility across therapeutic interventions for MDD. Identifying potential biological predictors of response will hopefully lead to a departure from the trial-and-error approach of MDD treatment, although several steps remain before declaring this achievement. Below, we will briefly define both resting EEG (rEEG) and TMS-EEG prior to presenting our systematic review of relevant findings.

### Resting EEG

Resting EEG (rEEG) indexes brain activity without stimulus presentation, typically *via* 64 electrodes distributed with the 10–20 system (Jasper, 1958). REEG frequency bands characterize the signal in delta to gamma domains (Niedermeyer, 1999). The low frequency delta band (<4 Hz) appears in stage 3 non-rapid eye movement sleep and is not typically seen in rEEG (Amzica and Steriade, 1998). Theta (4–8 Hz) is related to emotional processing and internal focus (Aftanas and Golocheikine, 2001; Aftanas et al., 2002). Frontal theta activity may reflect neurotransmission to and from the anterior cingulate cortex (ACC) (Asada et al., 1999), regions implicated in MDD (Spellman and Liston, 2020). The alpha (8–12 Hz) band appears during relaxation and is the most dominant band present in occipital or posterior regions (Niedermeyer, 1999). In MDD, the presence of alpha indicates brain regions with lower activity (Bruder et al., 1997). Beta (12–30 Hz) and gamma bands (>30 Hz) are considered “high-frequency”, reflecting alertness and concentration (Abhang et al., 2016) and attention and executive functioning (Freeman and Quiroga, 2013), respectively. Deciphering the relevance of frequency band activity may have potential for MDD response markers.

### TMS-EEG

Transcranial magnetic stimulation (TMS) non-invasively stimulates the brain, inducing electric currents in neurons *via* electromagnetic induction (i.e., Faraday's law) (Barker et al., 1985). The TMS stimulus is thought to act on inhibitory interneurons and results in the depolarization of pyramidal cells (Kobayashi and Pascual-Leone, 2003), which can be captured *via* EEG. The combination of TMS-EEG then provides an accurate window into the direct localized and downstream cortical effects of the TMS pulse, and can provide measurable output for cortical regions outside of the motor and somatosensory cortices. Single-pulse TMS-EEG produces TMS evoked potentials (TEPs) reflecting excitatory and inhibitory neurotransmission (Farzan et al., 2016) and can index both inter and intra-regional connectivity between cortico-cortical and cortico-subcortical areas (Daskalakis et al., 2012). Unlike rEEG, TMS-EEG allows for both direct stimulation and recording of output from the cortical region of interest. Both cortical responses at the stimulated region, as well as downstream effects can then be interpreted. These measures have identified cortical abnormalities in MDD, that may be used as markers of treatment response.

### Objectives of review

We conducted a formal narrative synthesis of the included studies, which focused on rEEG and TMS-EEG indexing the effects of antidepressant interventions (pharmacotherapy, brain stimulation, other therapies) on resulting outcomes (response or remission from a major depressive episode). The objectives were to: report changes in EEG measures of treatment; compare changes in EEG measures following treatment in responders and non-responders; report whether EEG measures at baseline predicted response.

## Methods

### Search strategy

PubMed and EMBASE were searched between January 1st, 2000 and December 31st, 2021 for publications studying treatment effects (i.e., pharmacotherapies and non-pharmacotherapies) on EEG (rEEG and TMS-EEG) in patients with MDD. Search terms are detailed in the Appendix. Results were filtered to only include human studies reported in English.

### Inclusion criteria

Studies included examined subjects with unipolar MDD (DSM-IV and DSM-5 criteria) who underwent antidepressant treatment in conjunction with EEG measures. Studies must have reported rEEG or TMS-EEG measures before, during, or post-treatment.

### Exclusion criteria

Case studies, review articles, protocols, posters, and conference abstracts were excluded (i.e., incorrect design). Studies reporting on animal populations, healthy subjects, bipolar depression, or conditions other than unipolar MDD were excluded (i.e., incorrect patient population). Non-therapeutic interventions were also excluded (i.e., incorrect intervention). Studies reporting antidepressant effects using techniques other than EEG (i.e. magnetoencephalography and electromyography), were excluded (i.e., incorrect EEG type). Sleep EEG, ictal EEG, neurofeedback studies, resting connectivity EEG, event-related EEG, and machine learning studies were excluded for focus and brevity (i.e., incorrect outcome). For the purpose of this review, *incorrect* was used to denote criteria that deemed to be out of scope.

### Data extraction

Two study authors (RS, DV) conducted an independent literature search using pre-defined inclusion and exclusion criteria following duplicate removal. Covidence (www.covidence.org), an internet-based software, facilitated screening and extraction. Following initial screening, eligible studies underwent full-text review. Conflicts between authors were resolved by discussion. Approved studies were then moved to data extraction.

### Quality of evidence assessment

Quality of evidence assessment was performed using the Grading of Recommendations Assessment, Development, and Evaluation (GRADE) working group methodology (Schünemann et al., 2008). Quality was marked with four levels: high, moderate, low, very low. High studies were randomized, double-blinded, and placebo-controlled; moderate were randomized without blinding; low were non-randomized with a placebo or control group; very low were non-randomized without a placebo or control group. Studies marked *Very low* were excluded to focus on higher quality, and reliable designs.

## Results

### Study selection

**Figure 1:**
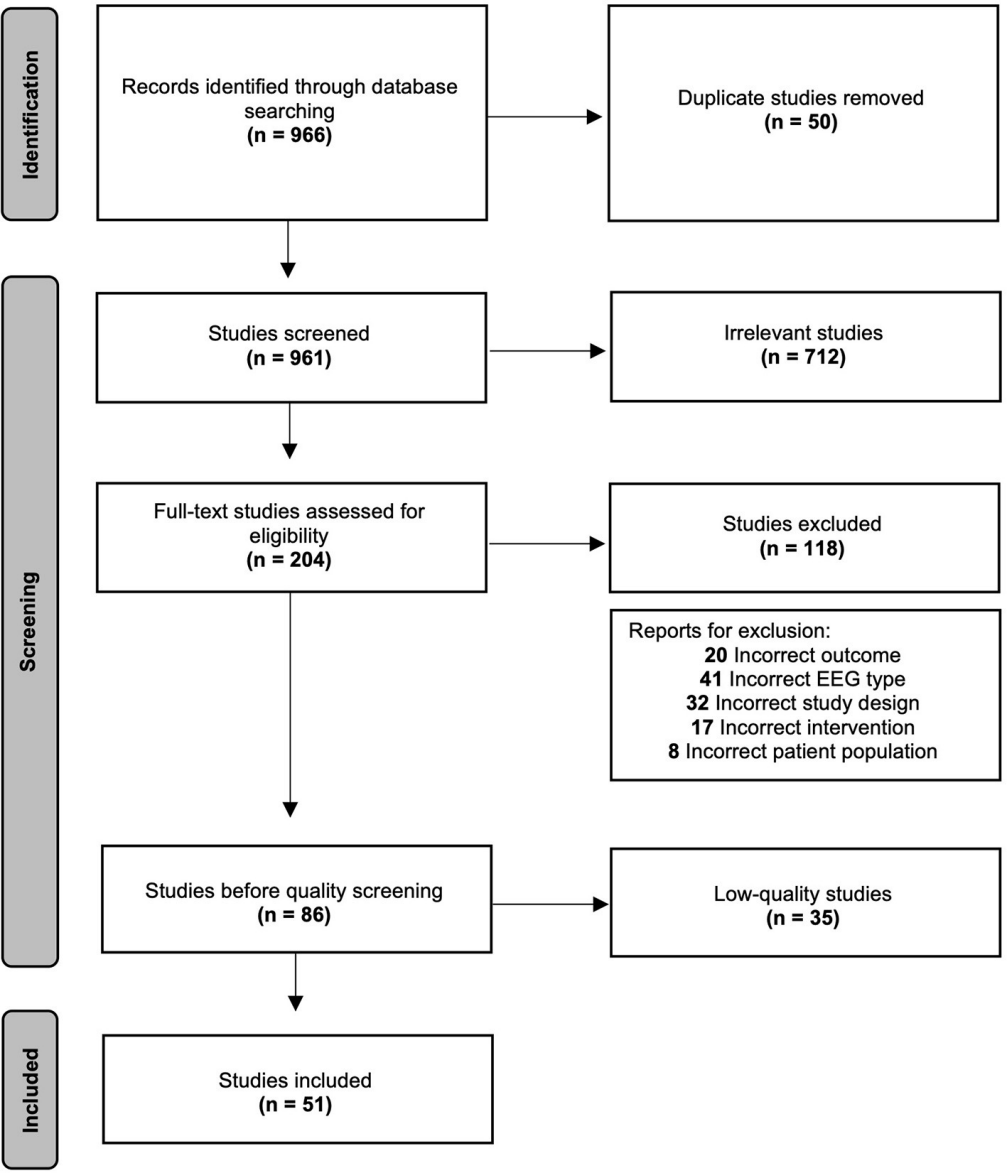
Preferred reporting items for systematic reviews and meta-analysis (PRISMA) flow diagram.

Figure 1 provides full information on the study selection process, using the Preferred Reporting Items for Systematic Reviews and Meta-Analysis (PRISMA).

### Included study characteristics

Search terms yielded 966 studies after applying filters, 916 after removing duplicates. Primary screening excluded 712 for irrelevance based on the abstract. Two hundred and four underwent full-text review. One hundred and eighteen were excluded for incorrect design, outcomes, EEG type, patient population, or intervention. For more details on reasons for exclusion, see Section Exclusion criteria. Thirty-five studies had a *Very low* quality assessment and were removed for brevity. Overall, 51 studies underwent qualitative synthesis.

Quality ratings are included in Tables 1, 2. After exclusion of *Very low* quality studies, the vast majority of studies were marked as *High*, followed by *Moderate* and *Low* ratings. The following sections are presented in order of decreasing quality rating.

**Table 1:** Resting EEG outcomes for antidepressant, brain stimulation, and other studies.

**rEEG mea- sure**	**References**	***n*** **(F)**	**EEG protocol (# of electrode; reference; electrode placement)**	**rEEG protocol (recording time; EO vs. EC; rEEG measure)**	**Recording period**	**Treatment type**	**Treatment name**	**Treatment protocol**	**Treatment length**	**Quality assessment**	**Outcome measure**	**Brain region**	**Change in measure following treatment**	**Association with response**
**Power analysis**														
	Szumska et al., 2021	20 (11)	64 electrodes; Cz reference; 10–20 system	3 min; EO and EC; α (8–13 Hz) band.	Baseline, post-treatment	Other therapies	Mindfulness-based cognitive therapy	2.5 h group sessions	8 sessions	Moderate	α asymmetry	FR	NS	NA
	McMillan et al., 2020	26 (13)	64 electrodes; FCz reference; NA	NA; NA; δ (1–4 Hz), θ (4–8 Hz), α (8–13 Hz), High β (28–40 Hz), Low γ (42–53 Hz), High γ (55–67 Hz) bands.	From time of infusion (few mins)	Other therapies	Ketamine or placebo	Ketamine 0.25 mg/kg.	1 infusion	High	θ power; High β power; Low γ power; High γ power; δ power; α power; Low β power	NA	↑; ↑; ↑; ↑; ↓; ↓; ↓	No; No; No; No; No; No; No
	Alexander et al., 2019	32 (27)	128 electrodes; Cz reference; 10–20 system	2 min; EC and EO; α (8–12 Hz) band.	Baseline, After 5 days of treatment, 4 week follow-up	Brain stimulation therapies	tACS or sham	10 Hz-tACS (*n* = 10) or 40 Hz-tACS (*n* = 11) or Active sham at 10 Hz (*n* = 11).	5 sessions	High	α power	FR	↓ Over LH (10 Hz- tACS only)	No
	Bailey et al., 2019	42 (23)	30 electrodes; CPz reference; NA	3 min; EO and EC; θ (4–8 Hz), α (8–13 Hz) bands.	Baseline, After 1-week of treatment, post-treatment	Brain stimulation therapies	rTMS	110% RMT, HF L-DLPFC or LF R-DLPFC or BL rTMS (CJ).	15 sessions	Low	θ power; α power	NA	NS; NS	No; No
	Cao et al., 2019	37 (32)	4 electrodes; A2 reference; NA	10 min; EC; δ (1–3.5 Hz), θ (4–7.5 Hz), lower α (8–10 Hz), upper α (10.5–12 Hz) bands.	Baseline, post-treatment	Other therapies	Ketamine or placebo	Ketamine 0.5 or 0.2 mg/kg.	1 infusion	High	Relative θ power; Relative α power	NA	↓; ↓	Yes; Yes
	Cook et al., 2019	16 (NA)	35 electrodes; Pz reference; 10–20 system	NA; EC; δ (0.5–4 Hz), θ (4–8 Hz), α (8–12 Hz), β (12–20 Hz) bands.	Baseline, Post-treatment	Brain stimulation therapies	sTMS or sham	sTMS (*n* = 10) or sham (*n* = 6).	6 weeks	High	Absolute power; Relative power	NA	NS; NS	NA; NA
	Leuchter et al., 2017	194 (124)	35 electrodes; Pz reference; NA	10 min; EC; δ + θ (2.5–8 Hz) or α (8–12 Hz) bands.	Baseline, After 1-week of treatment	Antidepressant pharmacotherapies	Escitalopram (SSRI) or placebo	Escitalopram (*n* = 143) 10 mg.	7 weeks	High	δ-θ power; α power	NA	↑ in SSRI group; ↓ in SSRI group	Yes; Yes
	Arns et al., 2016	655 (378)	26 electrodes; Average mastoid reference; 10–20 system	2 min; EO and EC; α (NA) band.	Baseline	Antidepressant pharmacotherapies	Escitalopram (SSRI) or Sertraline (SSRI) or Venlafaxine-XR (SNRI)	Escitalopram (*n* = 217) 10–20 mg. Sertraline (*n* = 234) 50–200 mg. Venlafaxine-XR (*n* = 204) 75–255 mg.	8 weeks	Moderate	α power	FR	↑ Right FR in SSRI group only.	Yes, for F only
	Arns et al., 2014	90 (49)	26 electrodes; Average mastoid reference; 10–20 system	2 min; EC and EO; α (7–13 Hz) band.	Baseline	Brain stimulation therapies	rTMS and psychotherapy	110% MT, HF L-DLPFC or LF R-DLPFC rTMS/	21 sessions	Low	α power	NA	NS	NA
	Gollan et al., 2014	37 (26)	20 electrodes; Average mastoid reference; 10–20 system	8 min; EC and EO; α (8–13 Hz) bands.	Baseline, Post-treatment	Other therapies	Behavioral Activation Treatment	CJ	16 sessions	Low	α asymmetry	FR	NS	No
	Jaworska et al., 2014	51 (28)	32 electrodes; Average mastoid reference; 10-10 system	3 min; EC; α (10.5–13 Hz) band.	Baseline, After 1-week of treatment	Antidepressant pharmacotherapies	Escitalopram (SSRI) or Bupropion (DRI) or SSRI + DRI	Escilatopram (*n* = 17) 10–40 mg. Bupropion (*n* = 16) 150–450 mg.	12 weeks	Moderate (without placebo-controlled)	α power	FR	↓ in SSRI group	Yes
	Widge et al., 2013	180 (NA)	4 electrodes; NA; NA	30 s; EO; α (8.5–12 Hz), β (12–20 Hz), θ (2–8.5 Hz) bands.	Baseline	Brain stimulation therapies	rTMS or sham	120% MT, HF L-DLPFC rTMS or sham.	6 weeks	Moderate (single-blinded)	α power; β power; θ power	NA	NA; NA; NA	NS; NS; NS
	Deslandes et al., 2010	20 (14)	20 electrodes; Linked ears reference; 10–20 system	8 min; NA; α (8–13 Hz) band.	Baseline, Post-treatment	Other therapies	Exercise + pharmacotherapy (Decided by physician)	Exercise group + pharmacotherapy (CJ) (*n* = 10) or pharmacotherapy only (CJ) (*n*=10)	1 year (2 exercise	Low	Absolute α power	NA	↓ pharmacotherapy group only	No
	Cook, 2002	51 (32)	35 electrodes; Pz reference; 10–20 system	NA; EC; θ (4–8 Hz) band.	Baseline, After 48 h, 1 week of treatment	Antidepressant pharmacotherapies	Fluoxetine (SSRI) or Venlafaxine (SNRI) or placebo	Fluoxetine 20 mg. Venlafaxine 37.5–150 mg.	8 weeks	High	Absolute or relative power	NA	NS	NA
	Knott et al., 2002	25 (NA)	21 electrodes; Linked-ears reference; 10–20 system	20 min; EC; δ (1.5–3.5 Hz), θ (3.5–7.5 Hz), α (7.5–12.5 Hz), β (12.5–25 Hz) bands.	Baseline, post-treatment	Antidepressant pharmacotherapies	Paroxetine (SSRI) or placebo	Paroxetine 20 mg.	6 weeks	Low	Absolute α power; Absolute β power; Relative δ power; Relative β power; Relative θ power; Relative α power	NA	↓; ↑; ↑; ↑; ↑; ↓	No; No; No; No; No; No
	Leuchter et al., 2002	51 ( 31)	35 electrodes; Pz reference; 10–20 system	NA; EC; δ (0.5–4 Hz), θ (4–8 Hz), α (8–12 Hz), β (12–10 Hz) bands.	Baseline, 1-week post placebo, After 2, 3, 8 weeks of treatment	Antidepressant pharmacotherapies	Fluoxetine (SSRI) or Venlafaxine (SNRI) or placebo	Fluoxetine (*n* = 24) 20 mg. Venlafaxine (*n* = 27) 37.5–150 mg.	7–8 weeks	High	Absolute δ power; Absolute θ power; Absolute α power; Absolute β power	NA	NS; NS; NS; NS	No; No; No; No
**Cordance**														
	de la Salle et al., 2020	46 (26)	32 electrodes; Common average reference; 10–10 system	3 min; EC; PF (Fpz, Fp2) and MRF (FZ, Fp1, F4, F8) θ (4–8 Hz) Cordance calculated.	Baseline, After 1-week of treatment	Antidepressant pharmacotherapies	Escitalopram (SSRI) or Bupropion (DRI) or SSRI + DRI	CJ	12 weeks	Moderate (without placebo-controlled)	θ cordance	PF, MRF	↓	Yes (+ Remission)
	Bailey et al., 2019	42 (23)	30 electrodes; CPz reference; NA	3 min; EO and EC; θ (4–8 Hz) Cordance calculated.	Baseline, After 1-week of treatment, post-treatment	Brain stimulation therapies	rTMS	110% RMT, HF L-DLPFC or LF R-DLPFC or BL rTMS (CJ)	15 sessions	Low	θ cordance	NA	NS	No
	Cao et al., 2019	37 (32)	4 electrodes; A2 reference; NA	10 min; EC; θ (4–7.5 Hz) Cordance calculated.	Baseline, post-treatment	Other therapies	Ketamine or placebo	Ketamine 0.5 or 0.2 mg/kg.	1 infusion	High	θ cordance	NA	↓	Yes
	Bares et al., 2015a	25 (20)	21 electrodes; FCz reference; 10–20 system	10 min; EC; PF (FP1, FP2, Fz) θ (4–8 Hz) Cordance calculated.	Baseline, After 1-week of treatment	Brain stimulation therapies	rTMS + placebo	100% MT, LF R-DLPFC rTMS	4 weeks	Moderate (without placebo-)	θ cordance	NA	↓	Yes
	Bares et al., 2015b	25 (20)	21 electrodes; FCz reference; 10–20 system	10 min; EC; PF (FP1, FP2, Fz) θ (4–8 Hz) Cordance calculated.	Baseline, After 1-week of treatment	Antidepressant pharmacotherapies	Venlafaxine (SNRI) + sham	~267 mg/day	4 weeks	Moderate (without placebo-controlled)	θ cordance	PF	↓	Yes
	Hunter et al., 2010a	72 (43)	35 electrodes; Pz reference; 10–20 system	NA; EC; MRF (FPz, Fz, FP2, AF2, F4) θ (4–8 Hz) Cordance calculated.	Baseline, 1-week post-placebo, After 48, 1, 2, and 4 h of treatment, post-treatment	Antidepressant pharmacotherapies	Fluoxetine (SSRI) or Venlafaxine (SNRI) or placebo	Fluoxetine (*n* = 13) 20 mg. Venlafaxine (*n* = 24) 150 mg.	8 weeks	High	θ cordance	MRF	↓	Associated with treatment-emergent suicidal ideation
	Hunter et al., 2010b	94 (58)	35 electrodes; Pz reference; 10–20 system	NA; EC; MRF (AF2, F4, F8, FP2, FPz, Fz) θ (4–8 Hz) Cordance calculated.	Baseline, After 48 h, 1-week of treatment	Antidepressant pharmacotherapies	Fluoxetine (SSRI) or Venlafaxine (SNRI) or placebo	Fluoxetine (*n* = 14) 20 mg. Venlafaxine (*n* = 35) 150 mg.	8 weeks	High	θ cordance	MRF	↓	Yes
	Cook et al., 2009	37 (23)	35 electrodes; Pz reference; 10–20 system	NA; EC; MRF (FPz, Fz, FP2, AF2, F4, F8) θ (4–8 Hz) Cordance calculated.	After 48 h, 1-week, 2 weeks of treatment	Antidepressant pharmacotherapies	Fluoxetine (SSRI) or Venlafaxine (SNRI) or placebo	Fluoxetine (*n* = 13) 20 mg. Venlafaxine (*n* = 24) 150 mg.	8 weeks	High	θ cordance	MRF	↓	Yes (+ Remission)
	Hunter et al., 2009	58 (NA)	35 electrodes; Pz reference; 10–20 system	NA; EC; PF (FP1, FPz, FP2) θ (4–8 Hz) Cordance calculated.	Baseline, 1-week post-placebo	Antidepressant pharmacotherapies	Fluoxetine (SSRI) or Venlafaxine (SNRI) or placebo	Fluoxetine (*n* = 13) 20 mg. Venlafaxine (*n* = 24) 150 mg.	8 weeks	High	θ cordance	PF	↓	Yes, during placebo lead-in in F only
	Hunter et al., 2006	51 (35)	35 electrodes; Pz reference; 10–20 system	NA; EC; θ (4–8 Hz) Cordance calculated.	Baseline, 1-week post-placebo, After 48, 1, 2, and 4 h of treatment, post-treatment	Antidepressant pharmacotherapies	Fluoxetine (SSRI) or Venlafaxine (SNRI) or placebo	Fluoxetine (*n* = 24) 20 mg. Venlafaxine (*n* = 27) 150 mg.	8 weeks	High	θ cordance	PF	↓	Yes, during placebo lead-in
	Cook, 2002	51 (32)	35 electrodes; Pz reference; 10–20 system	NA; EC; θ (4–8 Hz) Cordance calculated.	Baseline, After 48 h, 1 week of treatment	Antidepressant pharmacotherapies	Fluoxetine (SSRI) or Venlafaxine (SNRI) or placebo	Fluoxetine 20 mg. Venlafaxine 37.5–150 mg.	8 weeks	High	θ cordance	PF	↓	Yes
	Leuchter et al., 2002	51 (31)	35 electrodes; Pz reference; 10–20 system	NA; EC; θ (4–8 Hz) Cordance calculated.	Baseline, 1-week post-placebo, After 2, 3, 8 weeks of treatment	Antidepressant pharmacotherapies	Fluoxetine (SSRI) or Venlafaxine (SNRI) or placebo	Fluoxetine (*n* = 24) 20 mg. Venlafaxine (*n* = 27) 37.5–150 mg.	7–8 weeks	High	θ cordance	PF	↑ Placebo responders and ↓ medication responders	Yes; Yes
**Current density**														
	Pizzagalli et al., 2018	248 (160)	72 electrodes; Common average reference; NA	2 min; EC; θ current density calculated.	Baseline, After 1-week of treatment	Antidepressant pharmacotherapies	Sertraline (SSRI) or placebo	Sertraline (*n* = 121) ~200 mg.	8 weeks	High	θ current density	rACC	↑ (Non-specific for treatment group)	Yes
	Arns et al., 2015	655 (378)	26 electrodes; Average mastoid reference; 10–20 system	2 min; EC; θ current density calculated.	Baseline, post-treatment	Antidepressant pharmacotherapies	Escitalopram (SSRI) or Sertraline (SSRI) or Venlafaxine-XR (SNRI)	Escitalopram (*n* = 217) 10–20 mg. Sertraline (*n* = 234) 50–200 mg.Venlafaxine-XR (*n* = 204) 75–255 mg.	8 weeks	Moderate	θ current density	rACC, PF	↓ (More pronounced in TRD)	Yes
	Almeida Montes et al., 2015	74 (64)	32 electrodes; Average mastoid reference; 10–20 system	20 min; EC; α current density calculated.	Baseline, After 1- and 2-weeks of treatment, After 1,2, 6, 9, and 12 months of treatment	Antidepressant pharmacotherapies	Fluoxetine (SSRI)	Fluoxetine (SSRI) 20 mg during week 1, 40 mg from week 2- 1 year	1 year	Low	α current density	Occipital, Parietal, ACC, mOFC, thalamus, caudate nucleus	↓	No
	Jaworska et al., 2014	51 (28)	32 electrodes; Average mastoid reference; 10–10 system	3 min; EC; current density calculated.	Baseline, After 1-week of treatment	Antidepressant pharmacotherapies	Escitalopram (SSRI) or Bupropion (DRI) or SSRI + DRI	Escilatopram (*n* = 17) 10–40 mg. Bupropion (*n* =16) 150–450 mg.	12 weeks	Moderate (without placebo-controlled)	θ current density	rACC	↑ In SSRI + DRI group	Yes
	Hunter et al., 2013	22 (12)	36 electrodes; Pz reference; 10–20 system	20 min; EC; θ current density calculated.	5-weeks pre-treatment, immediately post-treatment (baseline)	Antidepressant pharmacotherapies	Sertraline (SSRI) or placebo	Sertraline 50–150 mg.	8 weeks	High	θ current density	rACC	↑	Yes
	Korb et al., 2011	72 (43)	36 electrodes; Pz reference; 10–20 system	20 min; EC; θ current density calculated.	Baseline	Antidepressant pharmacotherapies	Fluoxetine (SSRI) or Venlafaxine (SNRI) or placebo	Fluoxetine (*n* = 37) 150 mg. Venlafaxine (*n* = 35) 20 mg.	8 weeks	High	θ current density	rACC; mOFC	↑; NS	Yes; NA
	Tenke et al., 2011	41 (24)	67 electrodes; Average PO1 and PO2 references; NA	2 min; EC and EO; α current density calculated.	Baseline	Antidepressant pharmacotherapies	SSRI or SNRI or SSRI + NDRI	CJ	8–12 weeks	Low	α current density	NA	↑	Yes
	Narushima et al., 2010	43 (25)	19 electrodes; Linked ears reference; 10–20 system	20 min; EC; θ current density calculated.	Baseline, post-treatment	Brain stimulation therapies	rTMS or sham	110% MT, HF L-DLPFC rTMS (*n* = 32) or sham (*n* = 11).	2 weeks	Moderate	θ current density	sACC; rACC	↑; ↓	Yes; Yes
	Korb et al., 2009	72 (43)	36 electrodes; Pz reference; 10–20 system	20 min; EC; θ current density calculated.	Baseline	Antidepressant pharmacotherapies	Fluoxetine (SSRI) or Venlafaxine (SNRI) or placebo	Fluoxetine (*n* = 13) 150 mg. Venlafaxine (*n* = 24) 20 mg.	8 weeks	High	θ current density	rACC; mOFC	↑; ↑	Yes; Yes
	Mulert et al., 2007a	20 (13)	33 electrodes; Cz reference; 10–20 system	5 min; EC; θ current density calculated.	Baseline	Antidepressant pharmacotherapies	Citalopram (SSRI) or Reboxetine (NRI)	Citalopram (*n* = 11) 20–60 mg. Reboxetine (*n* = 7) 4–12 mg.	4 weeks	Moderate	θ current density	rACC; mOFC	↑; ↑	Yes; Yes
	Mulert et al., 2007b	20 (13)	33 electrodes; Cz reference; 10–20 system	5 min; EC; θ current density calculated.	Baseline	Antidepressant pharmacotherapies	Citalopram (SSRI) or Reboxetine (NRI)	Citalopram (*n* = 11) 20–60 mg. Reboxetine (*n* = 7) 4–12 mg.	4 weeks	Moderate	θ current density	rACC	↑	Yes
	Pizzagalli et al., 2001	18 (10)	28 electrodes; Average reference; 10–10 system	30 min; EC; θ current density calculated.	Baseline	Antidepressant pharmacotherapies	Nortriptyline (TCA)	Nortriptyline 50–150 ng/ml.	4–6 months	Low	θ current density	rACC	↑	Yes
**ATR**														
	Widge et al., 2013	180 (NA)	4 electrodes; NA; NA	30 s; EO; ATR calculated.	Baseline	Brain stimulation therapies	rTMS or sham	120% MT, HF L-DLPFC rTMS or sham.	6 weeks	Moderate (single-blinded)	ATR	NA	NS	NA
	Cook et al., 2013	67 (45)	4 electrodes; NA; NA	6 min and 2 min EO; EC; ATR calculated.	Baseline, After 1-week of treatment	Antidepressant pharmacotherapies	Escitalopram (SSRI) or Bupropion (DRI) or SSRI + DRI	Escilatopram 10 mg. Bupropion 300 mg.	13 weeks	Moderate	ATR	NA	↑	Yes (+Remission)
	Hunter et al., 2011	23 (15)	35 electrodes; Pz reference; 10–20 system	NA; EC; ATR calculated.	Baseline, post-treatment	Antidepressant pharmacotherapies	Fluoxetine (SSRI) or placebo	Fluoxetine (*n* = 13) 20 mg.	8 weeks	High	ATR	NA	↑ In SSRI group	Yes
	Leuchter et al., 2009b	220 (137)	2 electrodes; Fpz reference; NA	6 min EC and 2 min EO; ATR calculated.	Baseline, After 1-week of treatment	Antidepressant pharmacotherapies	Escitalopram (SSRI) or Bupropion (DRI) or SSRI + DRI	Escilatopram (*n* = 73) 10 mg. Bupropion (*n* = 73) 300 mg. Escilatopram + Bupropion (*n* = 74).	7 weeks	Moderate	ATR	NA	↑ In SSRI group	Yes (+Remission)
	Leuchter et al., 2009a	220 (137)	2 electrodes; Fpz reference; NA	6 min EC and 2 min EO; ATR calculated.	Baseline, After 1-week of treatment	Antidepressant pharmacotherapies	Escitalopram (SSRI) or Bupropion (DRI) or SSRI + DRI	Escilatopram (*n* = 73) 10 mg. Bupropion (*n* = 73) 300 mg. Escilatopram + Bupropion (*n* = 74).	7 weeks	Moderate	ATR	NA	↑ In SSRI group, ↓ in DRI group	Yes (+Remission); Yes
**Vigilance**														
	Ip et al., 2021	91 (66)	256 electrodes; Vertex reference; NA	3 min; EC and EO; EEG vigilance calculated using algorithm.	Baseline, post-treatment	Antidepressant pharmacotherapies	Escitalopram (SSRI) or Duloxetine (SNRI)	Escilatopram (*n* = 76) ~5–20 mg. Duloxetine (*n* =15) ~30–120 mg.	8 weeks	Low	Stage 0; Sub-Stage A2; Stage B; Sub-stage B1	NA	NS; NS; NS; ↑	No; No; No; Yes
	Sander et al., 2018	27 (17)	31 electrodes; NA; 10–20 system	15 min; EC; EEG vigilance calculated using algorithm.	Baseline, post-treatment	Other therapies	Partial Sleep Deprivation	Awake from 1 a.m. to 8 p.m.	1 session	Low	Mean Vigilance Value	NA	↓	Yes
	Schmidt et al., 2017	65 (33)	31 electrodes; Common average reference; 10–20 system	NA; NA; EEG vigilance calculated using algorithm.	Baseline, post-treatment	Antidepressant pharmacotherapies	Escitalopram (SSRI) or Mirtazapine (atypical) or other	CJ	4 weeks	Low	Stage 0; Sub-Stage A2; Stage B; Sub-stage B1	NA	↓; ↓; ↑; ↑	Yes; Yes; Yes; Yes
	Olbrich et al., 2016	1,008 (NA)	26 electrodes; Average mastoid reference; 10–20 system	3 min; EC; EEG vigilance calculated using algorithm.	Baseline, post-treatment	Antidepressant pharmacotherapies	Escitalopram (SSRI) or Sertraline (SSRI) or Venlafaxine-XR (SNRI)	Escitalopram (*n* = 198) 10–20 mg. Sertraline (*n* = 216) 50–200 mg.Venlafaxine-XR (*n* = 184) 75–225 mg.	8 weeks	Moderate	CNS arousal	NA	↓ SSRI only	Yes (+Remission)
**Normalizations and abnormalities**														
	van der Vinne et al., 2019b	453 (247)	26 electrodes; Average mastoid reference; 10–20 system	2 min; EO and EC; α asymmetry calculated.	Baseline, post-treatment	Antidepressant pharmacotherapies	Escitalopram (SSRI) or Sertraline (SSRI) or Venlafaxine-XR (SNRI)	Escitalopram (*n* = 136) 10–20 mg. Sertraline (*n* = 169) 50–200 mg. Venlafaxine-XR (*n* = 148) 75–225 mg.	8 weeks	Moderate	α asymmetry	FR	↑ Over RH in SSRI group	Yes, in F only
	van der Vinne et al., 2019a	57 (NA)	26 electrodes; Average mastoid reference; 10–20 system	2 min; EO and EC; Presence of abnormal EEG activity	Baseline, post-treatment	Antidepressant pharmacotherapies	Escitalopram (SSRI) or Sertraline (SSRI) or Venlafaxine-XR (SNRI) or other	Escitalopram (*n* = 19) 10–20 mg. Sertraline (*n* = 10) 50–200 mg. Venlafaxine-XR (*n* = 10) 75–225 mg.	8 weeks	Moderate	Normalization	NA	NA	Yes, associated with 5.2x likelihood of response to Sertraline
	Arns et al., 2017	622 (356)	26 electrodes; Average mastoid reference; 10–20 system	2 min; EC; Presence of abnormal EEG activity	Baseline	Antidepressant pharmacotherapies	Escitalopram (SSRI) or Sertraline (SSRI) or Venlafaxine-XR (SNRI)	Escitalopram 10–20 mg. Sertraline 50–200 mg. Venlafaxine-XR 75–255 mg.	8 weeks	Moderate	Epileptiform EEG;EEG slowing;α peak frequency	NA	NA NA; NA	Presence of epileptiform EEG and EEG slowing associated with ↓response in SSRI and SNRI group. Presence of slow α peak associated with response to Sertraline only.
**iAPF**														
	Bailey et al., 2019	42 (23)	30 electrodes; CPz reference; NA	3 min; EO and EC; iAPF calculated.	Baseline, After 1-week of treatment, Post-treatment	Brain stimulation therapies	rTMS	110% RMT, HF L-DLPFC or LF R-DLPFC or BL rTMS (CJ).	15 sessions	Low	iAPF	NA	NS	No
	Philip et al., 2019	83 (70)	2 electrodes; NA; 10–20 system	NA; NA; iAPF calculated.	Baseline	Brain stimulation therapies	sTMS or sham	sTMS (*n* = 42) or sham (*n* =41)/	10 weeks	High	iAPF	NA	↑	Yes
**Entropy**														
	Jaworska et al., 2017	36 (21)	32 electrodes; Average mastoid reference; 10-10 system	3 min; EC and EO; MSE calculated.	Baseline	Antidepressant pharmacotherapies	Escitalopram (SSRI) or Bupropion (DRI) or combination	Escilatopram (*n* = 11) ~30 mg. Bupropion (*n* = 14) ~360 mg.	12 weeks	Moderate (without placebo controlled)	MSE	NA	↓ At fine temporal scales and ↑ at coarser temporal scales	Yes
**Other**														
	Arns et al., 2014	90 (49)	26 electrodes; Average mastoid reference; 10–20 system	2 min; EC and EO; LZC calculated.	Baseline	Brain stimulation therapies	rTMS + psychotherapy	110% MT, HF L-DLPFC or LF R-DLPFC rTMS.	21 sessions	Low	LZC	NA	↑	Yes

*ACC, anterior cingulate cortex; ATR, antidepressant treatment response; BL, bilateral; CJ, treatment titrated according to clinical judgment; CR, central region; DLPFC, dorsolateral prefrontal cortex; DMPFC, dorsomedial prefrontal cortex; DRI, dopamine reuptake inhibitor; EC, eyes closed; ECT, electroconvulsive therapy; EO, eyes open; FR, frontal region; HF, high-frequency; iAPF, individualized α peak frequency; LF, low-frequency; LH, left-hemisphere; LZC, Lempel-Ziv Complexity; MAOI, monoamine oxidase inhibitor; MRF, midline and right frontal; MSE, multiscale entropy; MT, motor threshold; NRI, norepinephrine reuptake inhibitor; NS, non-significant; PF, pre-frontal; PR, parietal region; rACC, rostral anterior cingulate cortex; rAI, right anterior insula; RH, right-hemisphere; RMT, resting motor threshold; rTMS, repetitive transcranial magnetic stimulation; SCC, subgenual cingulate cortex; sgACC, subgenual anterior cingulate cortex; SGPFC, subgenual prefrontal cortex; SNRI, serotonin norepinephrine reuptake inhibitor; SSRI, selective serotonin reuptake inhibitor; sTMS, synchronized transcranial magnetic stimulation; tACS, transcranial altering current stimulation; TCA, tricyclic antidepressant; TRD, treatment-resistant depression; UL, unilateral*.

**Table 2:** TMS-EEG outcomes for brain stimulation studies.

**TMS-EEG measure**	**References**	***n*** **(F)**	**EEG protocol (# of electrode; reference; electrode placement)**	**TMS-EEG protocol (stimulation intensity, TMS stimulation site)**	**Recording period**	**Treatment Type**	**Treatment name**	**Treatment protocol**	**Treatment length**	**Quality assessment**	**Outcome measure**	**Brain region**	**Change in measure following treatment**	**Association with response**
**TEP**														
	Voineskos et al., 2021	30 (15)	64 electrodes; Common average reference; NA	120% RMT; Single-pulse TMS to DLPFC.	Baseline, post-treatment	Brain stimulation therapies	rTMS	120% RMT, BL rTMS (R-DLPFC 1 Hz + L-DLPFC 10 Hz) or UL rTMS (L-DLPFC 10 Hz) or sham	30 sessions	High	P60; N45; N100	GMFA	NS; ↓; ↓	No; No; Yes
	Eshel et al., 2020	33 (19)	64 electrodes; Common average reference; NA	120% RMT; Single-pulse TMS to left/right DLPFC, left VAN, left V1.	Baseline, post-treatment	Brain stimulation therapies	rTMS	120% RMT, L-DLPFC 10 Hz rRMS or sham	20 sessions	High	P30	Frontal and Parietal electrodes	↓	Yes
**Power analysis**														
	Hill et al., 2021	38 (19)	64 electrodes; Common average reference; 10–20 system	120% RMT; Single-pulse TMS to DLPFC, motor cortex.	Baseline, post-treatment	Brain stimulation therapies	MST or ECT	BL MST (F3 and F4) or BL ECT	~1,420 sessions	Low	δ power; θ power; α power	MC; DLPFC	MST: ↓ over DLPFC; ↓ over DLPFC; ↓ over DLPFC ECT: ↓ over MC and DLPFC;↓ over MC and DLPFC; ↓ over DLPFC	MST: No; No; Yes ECT: No; No; Yes
**Other**														
	Hadas et al., 2019	26 (17)	64 electrodes; Common average reference; NA	NA; Single-pulse TMS to left DLPFC.	Baseline, post-treatment	Brain stimulation therapies	rTMS	120% RMT, BL rTMS (R-DLPFC 1 Hz + L-DLPFC 10 Hz) or UL rTMS (L-DLPFC 10 Hz) or sham	3–6 weeks	High	SCD; SCS	NA	NS; ↓	NA; Yes

*BL, bilateral; ECT, electroconvulsive therapy; MC, motor cortex; MST, magnetic seizure therapy; RMT, resting motor threshold; rTMS, repetitive transcranial magnetic stimulation; SCD, significant current density; SCS, significant current scattering; TEP, TMS-evoked potential; UL, unilateral; DLPFC, dorsolateral prefrontal cortex*.

### Resting EEG studies

Forty-seven studies examined rEEG markers of treatment response to pharmacotherapies, brain stimulation therapies, and other therapies (Table 1). These studies reported quantitative rEEG measures such as power analysis (Cook, 2002; Knott et al., 2002; Deslandes et al., 2010; Widge et al., 2013; Gollan et al., 2014; Jaworska et al., 2014, p. 201; Leuchter et al., 2002, 2017; Arns et al., 2014, 2016; Alexander et al., 2019; Bailey et al., 2019; Cao et al., 2019; Cook et al., 2019; McMillan et al., 2020; Szumska et al., 2021), cordance (Cook, 2002; Leuchter et al., 2002; Hunter et al., 2006, 2009, 2010a,b; Cook et al., 2009; Bares et al., 2015a; Bailey et al., 2019; Cao et al., 2019; de la Salle et al., 2020), current density (Pizzagalli et al., 2001, 2018; Mulert et al., 2007a,b; Korb et al., 2009, 2011; Narushima et al., 2010; Tenke et al., 2011; Hunter et al., 2013, p. 201; Jaworska et al., 2014; Almeida Montes et al., 2015; Arns et al., 2015), a weighted combination of alpha and theta power compared over time (termed: antidepressant treatment response (ATR) index) (Leuchter et al., 2009a,b; Hunter et al., 2011; Cook et al., 2013; Widge et al., 2013), vigilance (Olbrich et al., 2016; Schmidt et al., 2017; Sander et al., 2018; Ip et al., 2021), normalizations and abnormalities (Arns et al., 2017; van der Vinne et al., 2019a,b), individualized alpha-peak frequency (iAPF) (Bailey et al., 2019; Philip et al., 2019), entropy (Jaworska et al., 2017), and other algorithms (Arns et al., 2014).

rEEG studies included a variety of pharmacotherapies, brain stimulation therapies, and other therapies. Pharmacotherapy studies used various dosages, schedules, and antidepressant classes of medication [serotonin-reuptake inhibitors (SSRI), serotonin-norepinephrine reuptake inhibitors (SNRI), tricyclic antidepressants (TCA), dopamine reuptake inhibitors (DRI), mirtazapine, or combinations]. Some used fixed dosages and treatment lengths, others followed naturalistic designs. Brain stimulation interventions included repetitive transcranial magnetic stimulation (rTMS), synchronized transcranial magnetic stimulation (sTMS), and transcranial alternating current stimulation (tACS). Treatment parameters varied by stimulation target, intensity, and number of treatments. Other therapies included IV ketamine, psychotherapies (i.e., mindfulness-based cognitive therapy, behavioral activation treatment), aerobic training, and partial sleep deprivation. Across treatment types, rEEG protocols varied in recording length, electrode placement, number of electrodes of interest, eyes closed vs. open and outcome measures. The following section will focus on reported rEEG measures by treatment type.

#### Power analysis

Frequency bands are computed by absolute or relative band power. Absolute band power measures all activity within a specific range, whereas relative band power expresses band power as a percentage of total signal power.

##### Antidepressant pharmacotherapies

Three high quality rated studies reported power analysis findings. One week of SSRI resulted in decreased relative alpha power and increased relative delta-theta (Leuchter et al., 2017). However, there were no significant results in patients randomized to SSRI, SNRI or placebo (Cook, 2002; Leuchter et al., 2002). One moderate quality rated study reported higher frontal alpha absolute power at baseline in SSRI, DRI or combination pharmacotherapy responders which decreased in the SSRI group after 1 week (Jaworska et al., 2014). Additionally, female SSRI responders and remitters showed greater baseline right-sided alpha power (Arns et al., 2016). In one low rated study in men only, both SSRI and placebo resulted in increased relative and absolute beta, but decreased alpha power after 6 weeks (Knott et al., 2002). Overall, alpha findings warrant more exploration, however, it remains unclear whether other frequency bands show promise due to the variability in these results.

##### Brain stimulation therapies

Two high-quality studies reported power analysis outcomes. Patients randomized to receive 10 Hz tACS for 5 sessions showed decreased frontal alpha power over the left hemisphere, which was not noted in patients who received 40 Hz or sham tACS (Alexander et al., 2019). In contrast, there were no changes in absolute or relative power bands following 6 weeks of sTMS (Cook et al., 2019). *One* moderately rated HF L-DLPFC rTMS study (Widge et al., 2013) reported no absolute or relative power differences between responders and non-responders at baseline. In low quality rated studies, no differences in alpha power appeared between responders and non-responders to HF L-DLPFC or LF R-DLPFC rTMS combined with psychotherapy (Arns et al., 2014). Further, there were no changes in theta or alpha power following 3 weeks of HF L-DLPFC, LF R-DLPFC, or combination rTMS (Bailey et al., 2019). To conclude, most brain stimulation studies did not find significant power analysis findings.

##### Other therapies

Two high-quality studies reported IV ketamine effects on power analysis (Cao et al., 2019; McMillan et al., 2020). Post-infusion, theta, high-beta, and gamma power increased, whereas delta, alpha, and low-beta power decreased. However, no relationship was found between any bands and antidepressant response (McMillan et al., 2020). In contrast, in an earlier study, decreases in relative theta and alpha power following IV ketamine infusion were associated with treatment response (Cao et al., 2019). One moderate quality rated study following 8 sessions of mindfulness-based cognitive therapy showed no change in frontal alpha power over either cortical hemisphere (Szumska et al., 2021). Two low-rated studies reported conflicting results. Absolute alpha power decreased following 1-year of pharmacotherapy alone, but not in patients receiving pharmacotherapy plus aerobic training (Deslandes et al., 2010). In contrast, there was no change in frontal alpha power following 16 sessions of behavioral activation therapy (Gollan et al., 2014). As such, decreased alpha power appears to consistently be associated with treatment response across diverse modalities of antidepressant intervention.

#### Cordance

Cordance is quantified by integrating absolute and relative EEG power measures, and is strongly associated with cerebral blood perfusion (Leuchter et al., 1994, 1999). Similar to functional neuroimaging, it is used to quantify abnormalities in brain activity, namely over dysregulated regions in MDD such as the frontal cortex (Hunter et al., 2007).

##### Antidepressant pharmacotherapies

Seven high quality rated studies reported cordance. Treatment response was associated with decreased prefrontal (PF) theta cordance after 48 h and 1 week of SSRI or SNRI treatment (Cook, 2002). Similar results in midline and right frontal regions emerged after 8 weeks of SSRIs or SNRIs (Cook et al., 2009; Hunter et al., 2010b). Interestingly, decreases in midline and right frontal theta cordance were also associated with medication-induced suicidal ideation (Hunter et al., 2010a). PF theta cordance decrease during placebo lead-in was also associated with greater response (Hunter et al., 2006), especially in female participants (Hunter et al., 2009), but the opposite trend was found for placebo responders (Leuchter et al., 2002). Two moderately rated studies examined theta cordance. One week after SNRI initiation, decreased PF theta cordance predicted greater response (AUC = 0.89) (Bares et al., 2015b). This relationship also appeared 1 week after initiation of SSRI, DRI, or combination, in both PF and midline and right frontal regions (de la Salle et al., 2020). Taken together, the above evidence demonstrates that decreased theta cordance after 1 week of pharmacotherapy may be a reliable measure of forthcoming antidepressant response.

##### Brain stimulation therapies

No high quality rated studies were found addressing cordance measures with regard to brain stimulation. *One* moderately rated study reported decreased PF theta cordance following high-frequency (HF) left dorsolateral prefrontal cortex (DLPFC) rTMS with placebo in treatment-resistant depression (TRD) (Bares et al., 2015b). Higher PF theta cordance at baseline was correlated with a greater reduction in symptoms following 4 weeks of rTMS, and PF theta cordance values decreased in responders 1 week post-treatment (Bares et al., 2015b). One low quality rated study following 3 weeks of HF L-DLPFC, LF R-DLPFC, or combination rTMS found no significant changes in theta cordance (Bailey et al., 2019). Despite this conflicting evidence, the moderately rated brain stimulation study is more in line with trends of decreased PF theta cordance that were noted with antidepressants.

##### Other studies

Only one study met criteria for this section, a high quality rated investigation which reported decreased theta cordance in responders after IV ketamine infusion (Cao et al., 2019). While singular, these findings echo the trend from pharmacotherapy and brain stimulation studies.

#### Current density

Current density quantifies rEEG activity, and is positively correlated with glucose metabolism (Pizzagalli et al., 2001). In MDD, abnormal metabolism levels in the rostral anterior cingulate cortex (rACC) and frontal regions have been related to symptom presentation (Martinot et al., 2011), and may be studied using current density.

##### Antidepressant pharmacotherapies

Four high quality rated studies reported theta current density. At baseline, responders showed higher rACC (SSRIs and SNRIs) and medial orbitofrontal cortex (mOFC) (SNRIs) (Korb et al., 2009; Hunter et al., 2013) theta current density, although another study did not replicate findings (Korb et al., 2011). Responders in both placebo and TCA groups showed elevated rACC at baseline and after 1 week of pharmacotherapy (Pizzagalli et al., 2018). Four moderate quality rated studies reported theta current density. In two, pharmacotherapy responders showed baseline elevated mOFC (NRIs and SSRIs) and rACC (NRIs) theta current density (Mulert et al., 2007a,b). DRI, but not SSRI responders, had higher baseline rACC theta current density. Responders showed increased rACC theta current density after 1 week of combination SSRI and DRI treatment (Jaworska et al., 2014). In contrast, at baseline and 8 weeks after SSRI or SNRI initiation, non-responders exhibited higher rACC and frontal theta current density, especially in non-responders with treatment resistant depression (Arns et al., 2015). Three low quality studies reported theta current density. One linked elevated baseline rACC theta current density with TCA response (Pizzagalli et al., 2001). Another, following 1 year of SSRIs, showed that patients exhibited lower alpha current density at each follow-up visit compared to healthy controls, despite some subjects reaching remission (Almeida Montes et al., 2015). In contrast, responders to SSRI, SNRI, or SSRI plus NDRI exhibited higher alpha current density at baseline compared to non-responders and healthy controls (Tenke et al., 2011). Given the above evidence, higher baseline theta current density shows clear promise in predicting pharmacotherapeutic response.

##### Brain stimulation therapies

One moderately rated randomized, sham-controlled HF L-DLPFC rTMS trial in patients with vascular depression recorded theta current density in the subgenual anterior cingulate cortex (sACC) and rACC (Narushima et al., 2010). At baseline, responders showed higher theta current density in sACC than non-responders, but no significant rACC findings were reported. No high or low quality rated studies were found in the literature review, but the single brain stimulation study reported findings consistent with antidepressant studies.

##### Other therapies

No studies reported on current density measures.

#### Antidepressant treatment response

The antidepressant treatment response (ATR) is a non-linear weighted combination of theta and alpha power, both relative and absolute, measured at baseline and 1 week after initiation (Leuchter et al., 2009a,b). However, it is unclear how the ATR directly reflects brain activity (Wade and Iosifescu, 2016).

##### Antidepressant pharmacotherapies

One high quality rated study reported higher ATR predicted both response and remission after 8 weeks of SSRIs (Hunter et al., 2011). Three moderately rated studies reported ATR levels following SSRI, DRI, or combination (Leuchter et al., 2009a,b; Cook et al., 2013). SSRI responders who underwent 7 weeks (Leuchter et al., 2009a,b) and 13 weeks (Cook et al., 2013) of treatment showed higher ATR values than non-responders, but findings were inconsistent for DRIs. Overall, higher ATR may predict response to some antidepressant pharmacotherapies.

##### Brain stimulation therapies

One moderately rated study reported no association between ATR and response following HF L-DLPFC rTMS (Widge et al., 2013).

##### Other therapies

No studies reported on the ATR measure.

#### Vigilance

EEG vigilance is a validated algorithm using rEEG frequency bands to quantify brain arousal into specific stages (Olbrich et al., 2009). Individuals with MDD typically show higher arousal patterns than healthy individuals (Hegerl et al., 2012).

##### Antidepressant pharmacotherapies

No high-quality rated studies were present in the literature review. One moderately rated study recorded vigilance as an index of central-nervous system arousal, and found decreased arousal in patients following 8-weeks of SSRIs, but not SNRI (Olbrich et al., 2016). Two low quality rated studies recorded vigilance following SSRIs, mirtazapine, SNRIs, or other medications (Schmidt et al., 2017; Ip et al., 2021). At baseline, responders had high vigilance in relaxed wakeful states and low vigilance in drowsy states, trends which were reversed after 4 weeks (Schmidt et al., 2017) and 8 weeks of treatment (Ip et al., 2021). Taken together, vigilance measures indicate that responders to various pharmacotherapies show high arousal at baseline that may be reversed following treatment.

##### Brain stimulation therapies

No studies reported on vigilance measures.

##### Other therapies

One low quality rated study which calculated mean vigilance values (MVV) as a measure of average brain arousal following partial sleep deprivation therapy showed that responders were characterized by lower MVV compared to non-responders (Sander et al., 2018).

#### Normalization and abnormalities

Abnormal EEG activity is characterized by slowing, epileptiform or paroxysmal activity, and alpha peak frequencies (APF) (Noachtar et al., 1999; Niedermeyer, 2005). In contrast, normalization occurs when abnormalities disappear or return to stable recording.

##### Antidepressant pharmacotherapies

Three moderately rated studies reported EEG normalization or abnormalities. Following SSRIs or SNRIs, epileptiform activity or slowing was negatively correlated with response (Arns et al., 2017). Slow APF was associated with SSRI response only (Arns et al., 2017). EEG normalization was noted in SSRI but not SNRI responders (van der Vinne et al., 2019a). Higher right-sided alpha power at baseline and after 8 weeks of SSRI was a stable marker associated with response in females, but not SNRI response (van der Vinne et al., 2019b). No high or low quality rated studies were present in the literature review. While there were inconsistencies, EEG abnormalities and normalization and stability may predict SSRI response.

##### Brain stimulation therapies

No studies reported on EEG normalizations and abnormalities.

##### Other therapies

No studies reported on EEG normalizations and abnormalities.

#### Individual alpha peak frequency

Individualized alpha peak frequency (iAPF) is used to quantify the average alpha power across frontal electrodes in eyes open and eyes closed conditions (Doppelmayr et al., 1998). It has been used to capture inter- and intra-individual differences in alpha frequency (Haegens et al., 2014).

##### Antidepressant pharmacotherapies

No studies reported on the iAPF measure.

##### Brain stimulation therapies

One high quality study demonstrated that higher iAPF at baseline was associated with response to 10 weeks of sTMS, compared to sham (Philip et al., 2019). In contrast, iAPF did not appear to have association with response after multiple therapeutic rTMS paradigms (HF L-DLPFC, LF R-DLPFC rTMS or BL rTMS) in a low quality rated study (Bailey et al., 2019). Thus, the predictive value of iAPF may be different depending on the type of brain stimulation delivered.

##### Other therapies

No studies reported on the iAPF measure.

#### Entropy

Multiscale entropy (MSE) quantifies brain signal variability over fine or coarse time scales (Costa et al., 2005), and may be used to study global and local connectivity disturbances in MDD (Jaworska et al., 2017).

##### Antidepressant pharmacotherapies

Only one moderately rated study recorded MSE with SSRI, DRI or combination pharmacotherapy. Responders were characterized by decreased baseline MSE at fine and increased MSE at coarser temporal scales (Jaworska et al., 2017).

##### Brain stimulation therapies

No studies reported on entropy measures.

##### Other therapies

No studies reported on entropy measures.

#### Other algorithms

The Lempel-Ziv Complexity (LZC) value quantifies the complexity of the EEG signal (see Bravi et al., 2011), to understand whether non-linear measures can better characterize cortical temporal patterns in MDD.

##### Antidepressant pharmacotherapies

No studies reported on the LCZ value.

##### Brain stimulation therapies

Only one low quality rated study reported on this measure. In patients undergoing HF L-DLPFC or LF R-DLPFC rTMS combined with psychotherapy (Arns et al., 2014), LZC increased from minute 1 to 2 of the baseline EEG in responders and decreased in non-responders. Further examination of LZC may further clarify its value as an EEG marker of brain stimulation response.

##### Other therapies

No studies reported on the LCZ value.

### TMS-EEG studies

No studies reported TMS-EEG outcome with antidepressant pharmacotherapies or other therapies. Four studies reported brain stimulation effects on TMS-EEG measures (Table 2). From the limited number of studies, TMS-EEG outcomes included TEP components (Eshel et al., 2020; Voineskos et al., 2021), power analysis (Hill et al., 2021), and other novel TMS-EEG algorithms (Hadas et al., 2019).

TMS-EEG studies mainly analyzed the effects of therapeutic rTMS, although the ECT and magnetic seizure therapy (MST) were also explored. Stimulation targets and duration/number of treatment sessions varied across studies. As well, diverse stimulation targets were applied in the investigatory TMS-EEG protocols. Due to the low number of studies, synthesis was limited.

#### TMS evoked potential components

TEPs over the motor cortex and DLPFC demonstrate replicable peaks (i.e., P30, N45, P60, N100) characterized by polarity and latency (Freeman and Quiroga, 2013). Several components have been linked to specific neurotransmitter receptor activity, including the P60 to glutamatergic receptor activity (Noda et al., 2017; Belardinelli et al., 2021), and N45 and N100 to gamma-aminobutyric acid (GABA) receptor activity (Farzan et al., 2013; Premoli et al., 2014; Rogasch et al., 2015).

##### Brain stimulation therapies

Two high quality rated investigations reported on the effects of a therapeutic course of rTMS on TEP components. Following 20 sessions of HF L-DLPFC rTMS, P30 amplitude decreased over the left frontal and parietal electrodes and was correlated with better clinical outcomes (Eshel et al., 2020). Similarly, the N45 and N100 amplitude decreased following 30 sessions of HF L-DLPFC or sequential bilateral rTMS (LF R-DLPFC rTMS followed by HF L-DLPFC rTMS), but there was no change to P60 (Voineskos et al., 2021). As well, the N100 decrease was related to improved depression symptoms post-treatment (Voineskos et al., 2021). Given the different components reported, high-quality replication studies are needed to elucidate the predictive ability of the P30, N45, and N100.

#### Power analysis

TMS-EEG power analysis is very similar to resting EEG power analysis, with frequency bands defined from alpha to theta.

##### Brain stimulation therapies

One low-quality rated study reported power analysis from TMS-EEG at baseline and after MST or ECT for TRD (Hill et al., 2021). Both treatments resulted in decreased delta and theta power over DLPFC (Hill et al., 2021). However, only ECT resulted in reduced alpha power over the DLPFC, and decreased delta and theta power over motor cortex (Hill et al., 2021). To this end, it is possible that MST effects were localized whereas ECT effects appear generalized over the cortex. As well, combined ECT and MST datasets showed a relationship between reduced alpha power and depression symptom improvements following treatment.

#### Novel TMS-EEG algorithms

Significant current density (SCD) and significant current scatter (SCS) are measures of subgenual cingulate cortex (SGC) excitability, and DLPFC-SGC effective connectivity, respectively (Hadas et al., 2019). Hyperactivity of the SGC and DLPFC-SGC connectivity have repeatedly been implicated in the pathophysiology of MDD (Mayberg et al., 1999).

##### Brain stimulation therapies

One high quality rated study examined SCS and SCD in patients with TRD. Here, the effects of HF L-DLPFC, sequential bilateral rTMS (LF R-DLPFC rTMS followed by HF L-DLPFC rTMS) or sham for 3–6 weeks were compared (Hadas et al., 2019). After active rTMS, SCS change and change in depression severity were positively correlated (Hadas et al., 2019). Further, after active rTMS, both P60 and P200 TEP component SCD decreased (Hadas et al., 2019). Given these results, TMS-EEG connectivity measures should be further explored in relation to treatment response.

## Discussion

We have presented a synthesis of existing literature focused on EEG markers of treatment response in MDD. In studies focused on rEEG markers, both theta cordance and theta current density consistently show potential as predictors of response for multiple modalities of MDD treatment. Decreased prefrontal theta cordance 1-week post-treatment was robust in predicting pharmacotherapy response, regardless of antidepressant medication class. The same trend was seen in higher quality brain stimulation and other therapy studies. Thus, theta cordance appears to be a reliable measure, especially for pharmacotherapies, perhaps in part due to the larger volume of studies focused on this intervention. Additionally, higher baseline theta current density may also have predictive value in pharmacotherapy response, with less existing evidence for brain stimulation interventions. In antidepressant studies, these findings were noted in rACC (Pizzagalli et al., 2001; Mulert et al., 2007b; Korb et al., 2009; Hunter et al., 2013; Jaworska et al., 2014) and mOFC (Mulert et al., 2007b; Korb et al., 2009). In contrast, alpha current density showed inconsistent value (Tenke et al., 2011; Almeida Montes et al., 2015). To this end, the replication of theta current density across higher-rated quality studies reinforces its potential as a predictive measure. These findings are encouraging given the biological implications of the theta band in MDD. Theta is usually most prominent in fronto-central regions, specifically the ACC and frontal cortex, both shown to be hypoactive in MDD (Asada et al., 1999). As theta is thought to represent drowsiness and low levels of cortical activation (Kropotov, 2016), it follows that this marker may hold high promise in our understanding of MDD. Thus, we encourage future biomarker guided clinical trials to verify theta markers of treatment response, specifically theta current density and theta cordance, as both show high potential to serve clinical utility in the treatment of MDD.

When examining power analysis, decreases in alpha power were the most consistently reported following treatment with SSRI (Knott et al., 2002; Jaworska et al., 2014; Leuchter et al., 2017), 10 Hz-tACS (Alexander et al., 2019), and IV Ketamine (Cao et al., 2019; McMillan et al., 2020). Thus, this measure may serve as a broad marker across treatment types, although further high-quality replication studies are needed. The remaining frequency bands (delta, theta, beta, and gamma) were reported in limited studies, with variable treatment types and inconsistent findings. Nevertheless, future focus on the alpha band is warranted given its ability to reflect inactivity of brain regions in MDD (Bruder et al., 1997). Previous research has linked the left frontal cortex to hypoactivity, reflected by high alpha power and reduced approach behavior (i.e., positive emotions) (Davidson, 1992). In contrast, the opposite trend is found over the right frontal cortex, reflected by low alpha power and increased withdrawal behavior (i.e., negative emotions). Taken together, the linkage between alpha power, cortical activity, and behavioral manifestations in MDD indicate the potential of this measure being extended to help guide treatment. Alpha band guided treatments have already proved useful in novel closed-loop neuromodulation techniques (Zrenner et al., 2016, 2020). Alpha oscillation-synchronized rTMS appears to improve treatment efficacy and may prove useful in personalizing therapeutic rTMS parameters (Zrenner et al., 2016, 2020). Phase synchronization has also been explored with the theta rhythm in healthy subjects, but requires replication in MDD populations undergoing therapeutic rTMS as a method for novel personalized treatments (Gordon et al., 2021). Additionally, sleep EEG power analysis may prove fruitful in identifying frequency band markers of MDD treatment response. Since the brain evidently behaves differently in wakeful and sleep states, especially for lower frequency bands, there is likely added value in exploring these markers in patients undergoing MDD interventions during sleep (Olbrich and Arns, 2013).

There were inconsistent findings across multiple treatment modalities with the remaining rEEG measures. First, higher ATR was replicated in responders or remitters to SSRIs (Leuchter et al., 2009a; Hunter et al., 2011), but was inconsistent for other medication classes such as DRIs (Leuchter et al., 2009b; Hunter et al., 2011), and in brain stimulation (Widge et al., 2013). Second, EEG abnormality measures were inconsistent, possibly due to high variability in selected outcome measures. SSRI response favored low EEG abnormalities and higher stability at baseline, but these were not predictive of SNRI response (Arns et al., 2017; van der Vinne et al., 2019a,b). Third, the different vigilance outcomes reported hindered the synthesis of results, but most studies reported that responders to various pharmacotherapies showed high brain arousal at baseline (Olbrich et al., 2016; Schmidt et al., 2017; Ip et al., 2021). Finally, very few studies reported iAPF (Bailey et al., 2019; Philip et al., 2019), entropy (Jaworska et al., 2017), and LCZ (Arns et al., 2014). While promise may remain within these measures, clear future directions cannot be gleaned from this synthesis of the literature.

In contrast, there were relatively few TMS-EEG studies to review, and all were focused on brain stimulation. Of the four studies included, three were high-quality, reflecting the high promise of TMS-EEG as a repository for neurophysiological biomarkers across brain stimulation modalities. rTMS appears to modulate DLPFC-SGC connectivity in parallel with improvements in MDD symptoms (Hadas et al., 2019). Additionally, better clinical outcomes were associated with decreased P30 amplitude (Eshel et al., 2020), and N100 amplitude (Voineskos et al., 2021) following active HF L-DLPFC and BL rTMS. While the biological association of the P30 is still unknown, the N100 seems to be linked to GABAergic receptor activity (Farzan et al., 2013; Premoli et al., 2014; Rogasch et al., 2015), a neurotransmitter highly implicated in the pathophysiology of MDD (Luscher et al., 2011). One low-quality study reported that decreased alpha power following MST or ECT was related to clinical response (Hill et al., 2021), adding to rEEG evidence of alpha band predictive power. There were three additional articles from our search that explored TMS-EEG effects following MST and ECT, however, they were deemed very *Low quality* and reported different outcomes, limiting synthesis (Casarotto et al., 2013; Miyauchi et al., 2019; Hadas et al., 2020). Overall, the high quality findings indicate that TMS-EEG measures may serve as MDD markers of response in the future.

There were some limitations to the literature reviewed. While non-linear rEEG quantitative algorithms (i.e., theta cordance, theta current density) show higher predictive value than traditional linear metrics, in part due to the non-linear behavior of brain function (Elbert et al., 1994), a recent meta-analysis calls the reliability of these indices into question, given the lack of direct replication studies and under-reporting of negative results (Widge et al., 2019). Further, evidence of biological linkages between rEEG measures and MDD symptomatology should also be verified using other modalities (i.e., imaging) or correlational studies before implementing these markers into clinical practice. A potential way of relieving these issues is by exploring novel computational and modeling approaches, which have gained traction. Cross-validated machine learning combining rEEG and mood measures show promise in distinguishing rTMS responders (Bailey et al., 2019). As well, a recent machine learning rEEG study differentially predicted response to SSRIs vs. low-frequency rTMS (Wu et al., 2020), which has generated much discussion on generalizability (Michel and Pascual-Leone, 2020; Nilsonne and Harrell, 2021; Wu et al., 2021). As such, while traditional rEEG measures are well documented, future studies may benefit from focusing on more complex indices.

A potential future direction of TMS-EEG markers may be in predicting specific MDD symptom improvements, as was shown by TMS-EEG indicators of suicidal ideation remission following MST (Sun et al., 2016). As well, combining TMS-EEG measures with other predictors of treatment response, such as rEEG outcomes or symptom presentation, using machine learning is another promising avenue that may serve clinical utility (Wu et al., 2020). Theta-burst stimulation (TBS), a novel rTMS therapy that produces significant antidepressant effects in patients with TRD (Blumberger et al., 2018), provides a further field for marker exploration with TMS-EEG. Notably, TBS builds on the concept of theta-gamma coupling, first proposed in animal models (Larson et al., 1986), a potential method of neural communication between brain regions thought to underly the basis of learning and memory (Lisman and Jensen, 2013). Taken together, TMS-EEG time-frequency analysis may be used to examine this theory by measuring changes in frequency power markers before and after TBS. Overall, TMS-EEG offers a novel area for discovery, offering replicable indices of cortical reactivity and connectivity that should be explored by future studies.

This review calls for more placebo-controlled, high-powered, replication studies to identify response markers for MDD treatments. Future EEG studies focusing on brain stimulation and novel therapeutics may lead to further understanding of neurophysiological treatment effects. We suggest a focus on TMS-EEG, given its potential to specifically target brain regions relevant to MDD. Specifically, emphasis on automation of TMS-EEG techniques and outcomes may eliminate variability in results, which can allow for more widespread clinical use in the future. In addition, rEEG has been more extensively studied than other methods, but further work is needed and the highest yield results are likely to emerge from the theta and alpha frequency markers defined above. Given the minimal cost associated with EEG, the potential for recordings to be distilled to a few electrodes and performed in community labs, it allows for far reaching real world clinical utility if such a treatment marker is identified. Importantly, improving predictions of treatment response, has the potential to spare patients and our healthcare system the burden of undergoing ineffective therapies, which would be of great clinical and scientific benefit.

## Data availability statement

The original contributions presented in the study are included in the article/supplementary material, further inquiries can be directed to the corresponding author.

## Author contributions

RS and DV conceived and designed the study, carried out the search, screening, data extraction, quality assessment, and drafted the manuscript. DV checked the inclusion/exclusion criteria. DMB, RC, and TKR critically revised the manuscript for intellectual content and reviewed the drafted manuscript. All authors contributed to critical revisions of the manuscript and gave final approval of the version to be published.

## Funding

RS was supported by an Ontario Graduate Scholarship. This work was also supported in part by an Academic Scholars Award from the Department of Psychiatry, University of Toronto, awarded to DV.

## Conflict of interest

TKR has received research support from Brain Canada, Brain and Behavior Research Foundation, BrightFocus Foundation, Canada Foundation for Innovation, Canada Research Chair, Canadian Institutes of Health Research, Center for Aging and Brain Health Innovation, National Institutes of Health, Ontario Ministry of Health and Long-Term Care, Ontario Ministry of Research and Innovation, and the Weston Brain Institute. TKR also received for an investigator-initiated study in-kind equipment support from Newronika, and in-kind research online accounts from Scientific Brain Training Pro, and participated in 2021 in an advisory board for Biogen Canada Inc. DMB receives research support from CIHR, NIH, Brain Canada and the Temerty Family through the CAMH Foundation and the Campbell Research Institute. He received research support and in-kind equipment support for an investigator-initiated study from Brainsway Ltd., and he has been the site principal investigator for three sponsor-initiated studies for Brainsway Ltd. He also receives in-kind equipment support from Magventure for investigator-initiated studies. He received medication supplies for an investigator-initiated trial from Indivior. He has participated in Scientific Advisory Board for Welcony Inc. DV holds the Labatt Family Professorship in Depression Biology, a University Named Professorship at the University of Toronto. She receives research support from CIHR, the Center for Addiction and Mental Health (CAMH) and the Department of Psychiatry at the University of Toronto. DV declares no biomedical interests or conflicts. The remaining authors declare that the research was conducted in the absence of any commercial or financial relationships that could be construed as a potential conflict of interest.

## Publisher's note

All claims expressed in this article are solely those of the authors and do not necessarily represent those of their affiliated organizations, or those of the publisher, the editors and the reviewers. Any product that may be evaluated in this article, or claim that may be made by its manufacturer, is not guaranteed or endorsed by the publisher.

## Appendix

Appendix 1. PubMed Search Strategy (“Depressive Disorder, Major/therapy”[Mesh]) OR (“Depression/therapy”[MAJR]) OR (“Antidepressive Agents/therapeutic use”[MAJR])) AND ((EEG) OR (electroencephalography) OR (TMS-EEG) OR (TMS)) Filters: English, Human Date range: 2000-2021.

Appendix 2. EMBASE Search Strategy (major depressive disorder) AND (antidepressant agent OR ECT OR transcranial magnetic stimulation OR repetitive transcranial magnetic stimulation) AND (electroencephalography) Filters: English, Human Date range: 2000-2021.
